# Supplementary material for: RYK Gene Expression Associated with Drug Response Variation of Temozolomide and Clinical Outcomes in Glioma Patients
Source: Pharmaceuticals (Basel). 2023 May 10;16(5):726. doi: 10.3390/ph16050726 (PMC10220703; doi:10.3390/ph16050726)
Supplement: Supplementary file 1 [file pharmaceuticals-16-00726-s001.zip › pharmaceuticals-2341653-supplementary.pdf]

pharmaceuticals

MDPI

Table S1. Minor Allele Frequency for SNP rs4470517.

Global	Study-wide	5008	G=0.1565	A=0.8435
African	Sub	1322	G=0.2163	A=0.7837
East Asian	Sub	1008	G=0.0050	A=0.9950
Europe	Sub	1006	G=0.2962	A=0.7038
South Asian	Sub	978	G=0.046	A=0.954
American	Sub	694	G=0.216	A=0.784

* 1000Genomes.BioProject ID: PRJEB6930

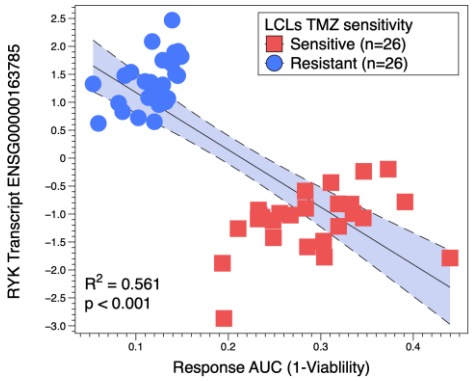

Figure S1. Correlation of Baseline *RYK* mRNA expression and cell viability regression and Pearsons correlation.

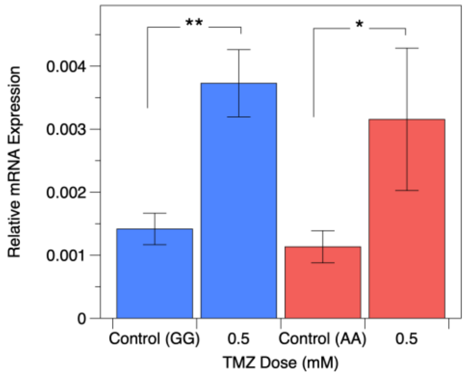

Figure S2. Drug-induced gene expression of *RYK* in LCL's. Transcriptional activity of *RYK* in LCL's measured following 72 h treatment with TMZ at the median concentration 0.5mM used in the cell viability screening. *p-value=0.01, **pvalue= 0.001

pharmaceuticals MDPI

Table S2. Predict SNP Outcomes

Genomic Position	rsID	REF	ALT	Genome Region	PredictSNP			CADD		
					Estimated effect	Score given by tool	Estimated expected accuracy	Estimated effect	Score given by tool	Estimated expected accuracy
chr3:134158269-134158269	rs56088702	A	G	intronic	deleterious	0.48902634	0.91	deleterious	11.39	0.67
chr3:134163307-134163307	rs4470517	G	A	intronic	deleterious	1	0.97	deleterious	19.8	0.96
chr3:134158726-134158726	rs4854617	C	A	intronic	neutral	-1	0.88	neutral	2.507	0.81
chr3:134158726-134158726	rs4854618	C	T	intronic	neutral	-0.33387833	0.74	neutral	1.941	0.88

DANN			FATHMM			FunSeq			GWAVA		
Estimated effect	Score given by tool	Estimated expected accuracy	Estimated effect	Score given by tool	Estimated expected accuracy	Estimated effect	Score given by tool	Estimated expected accuracy	Estimated effect	Score given by tool	Estimated expected accuracy
neutral	0.731191773	0.62	deleterious	0.98137	0.97	-	0.805443817	0.45	deleterious	0.55	0.8
deleterious	0.861943513	0.69	deleterious	0.82719	0.82	deleterious	0.995741679	0.68	deleterious	0.54	0.78
neutral	0.423580938	0.82	neutral	0.08253	0.89	neutral	0.187740611	0.83	neutral	0.3	0.68
deleterious	0.905213638	0.82	neutral	0.14228	0.91	neutral	0.187740611	0.83	neutral	0.3	0.68

*DANN (deleterious annotation of genetic variants using neural networks). Quang, D.; Chen, Y.; Xie, X. DANN: a deep learning approach for annotating the pathogenicity of genetic variants. Bioinformatics 2015, 31, 761–763, doi:10.1093/bioinformatics/btu703.

**FATHMM (Functional Analysis through Hidden Markov Models) . Shihab, H.A.; Gough, J.; Mort, M.; Cooper, D.N.; Day, I.N.M.; Gaunt, T.R. Ranking non-synonymous single nucleotide polymorphisms based on disease concepts. Hum Genomics 2014, 8, 11, doi:10.1186/1479-7364-8-11.

***GWAVA (Genome Wide Annotation of Variants). Ritchie, G.R.S.; Dunham, I.; Zeggini, E.; Flicek, P. Functional annotation of noncoding sequence variants. Nat. Methods 2014, 11, 294–296, doi:10.1038/nmeth.2832.

****FunSeq . Bahcall, O. FunSeq for cancer genomics. Nat. Genet. 2013, 45, 1273–1273, doi:10.1038/ng.2819.

*****CADD(Combined annotation-dependent depletion) Rentzsch, P.; Schubach, M.; Shendure, J.; Kircher, M. CADD-Splice-improving genome-wide variant effect prediction using deep learning-derived splice scores. Genome Med. 2021, 13, 31, doi:10.1186/s13073-021-00835-9.

*******PredictSNP2 Bendl, J.; Musil, M.; Štourač, J.; Zendulka, J.; Damborský, J.; Brezovský, J. Predictsnp2: A unified platform for accurately evaluating SNP effects by exploiting the different characteristics of variants in distinct genomic regions. PLoS Comput. Biol. 2016, 12, e1004962, doi:10.1371/journal.pcbi.1004962.

pharmaceuticals

MDPI

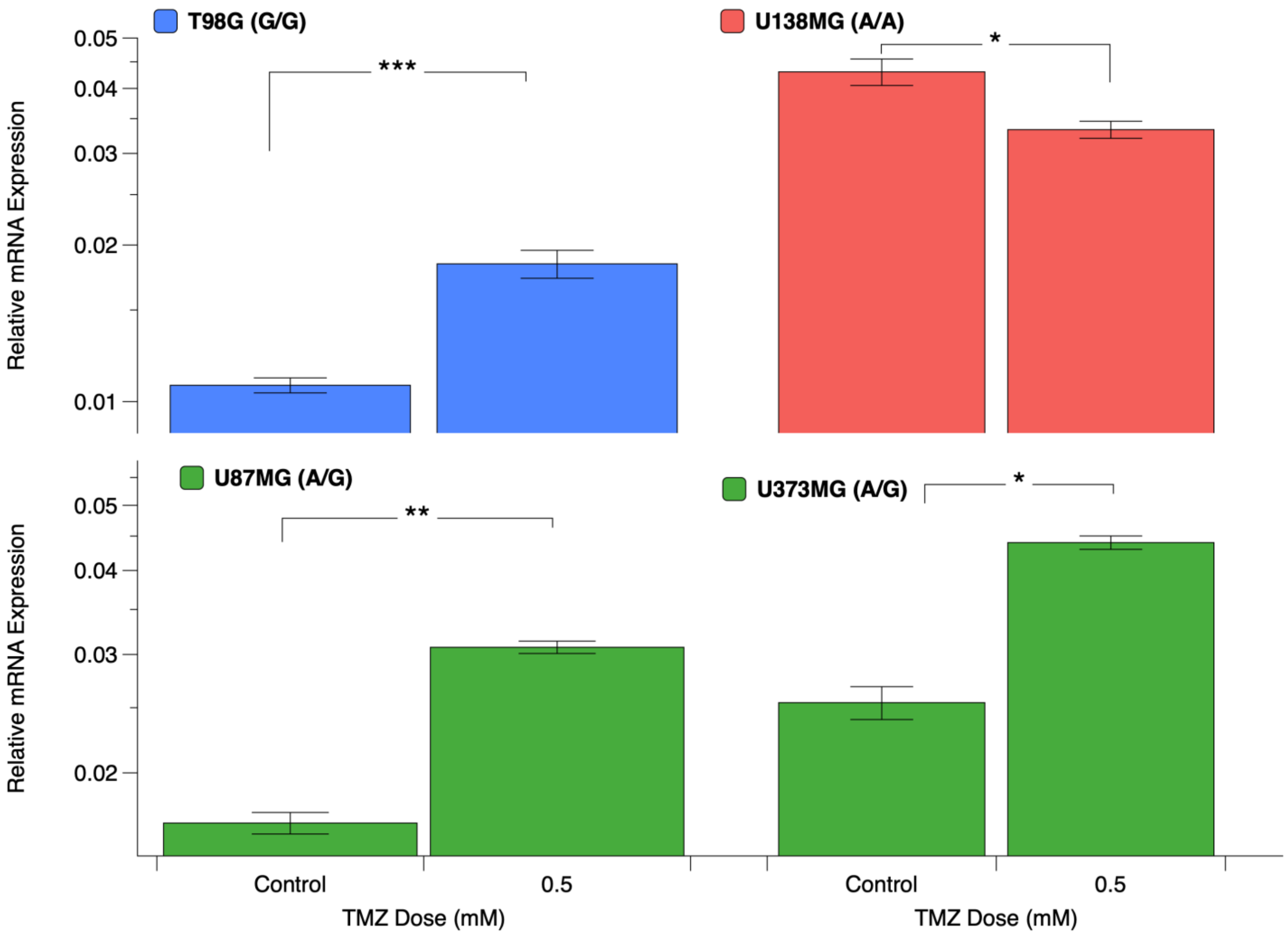

Figure S3. Drug-induced gene expression of *RYK* in glioblastoma cell lines. Transcriptional activity of *RYK* in 4 glioblastoma cancer cell lines measured following 72 h treatment of TMZ with at the median concentrations;0.75mM,0.5mM and 0.39mM used in the cell viability screening. *p < 0.05, ** p< 0.005,***p <0.0005

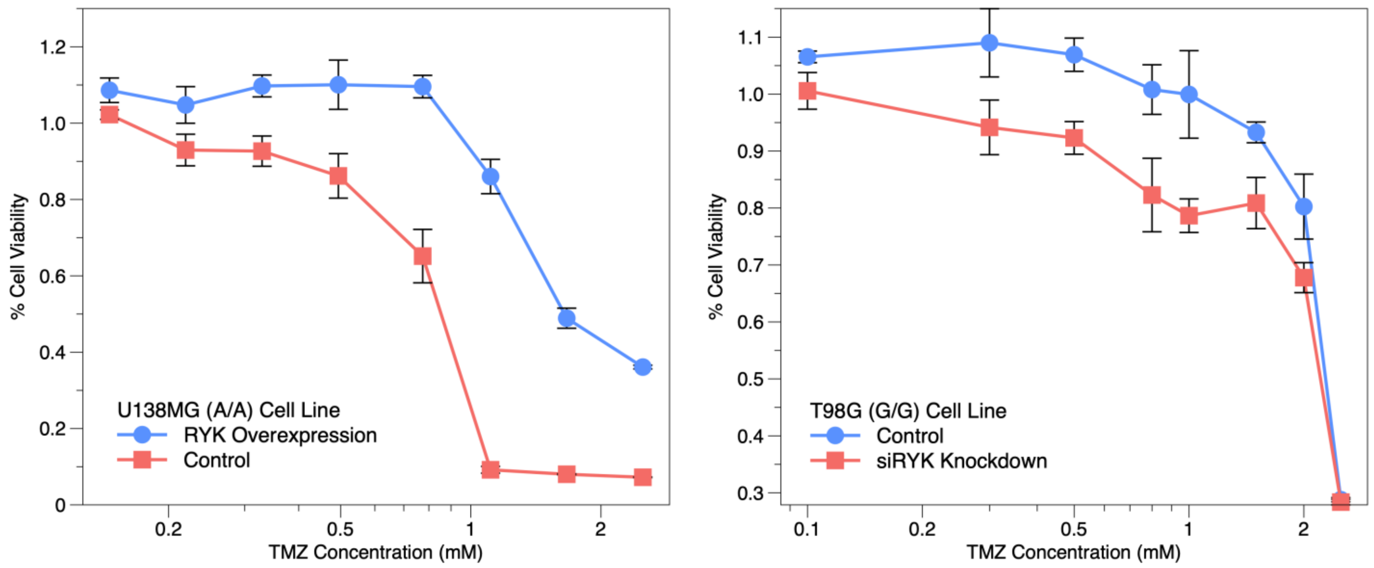

(**a**) (**b**)

Figure S4. glioma cell line *RYK* overexpression and knockdown. **(a)** U138MG *RYK* overexpression TMZ dose-response profile. **(b)** T98G *RYK* siRNA knockdown TMZ dose-response profile. Concentrations are on the log10 scale on the X-axis. Error bars represent the standard error of the mean.

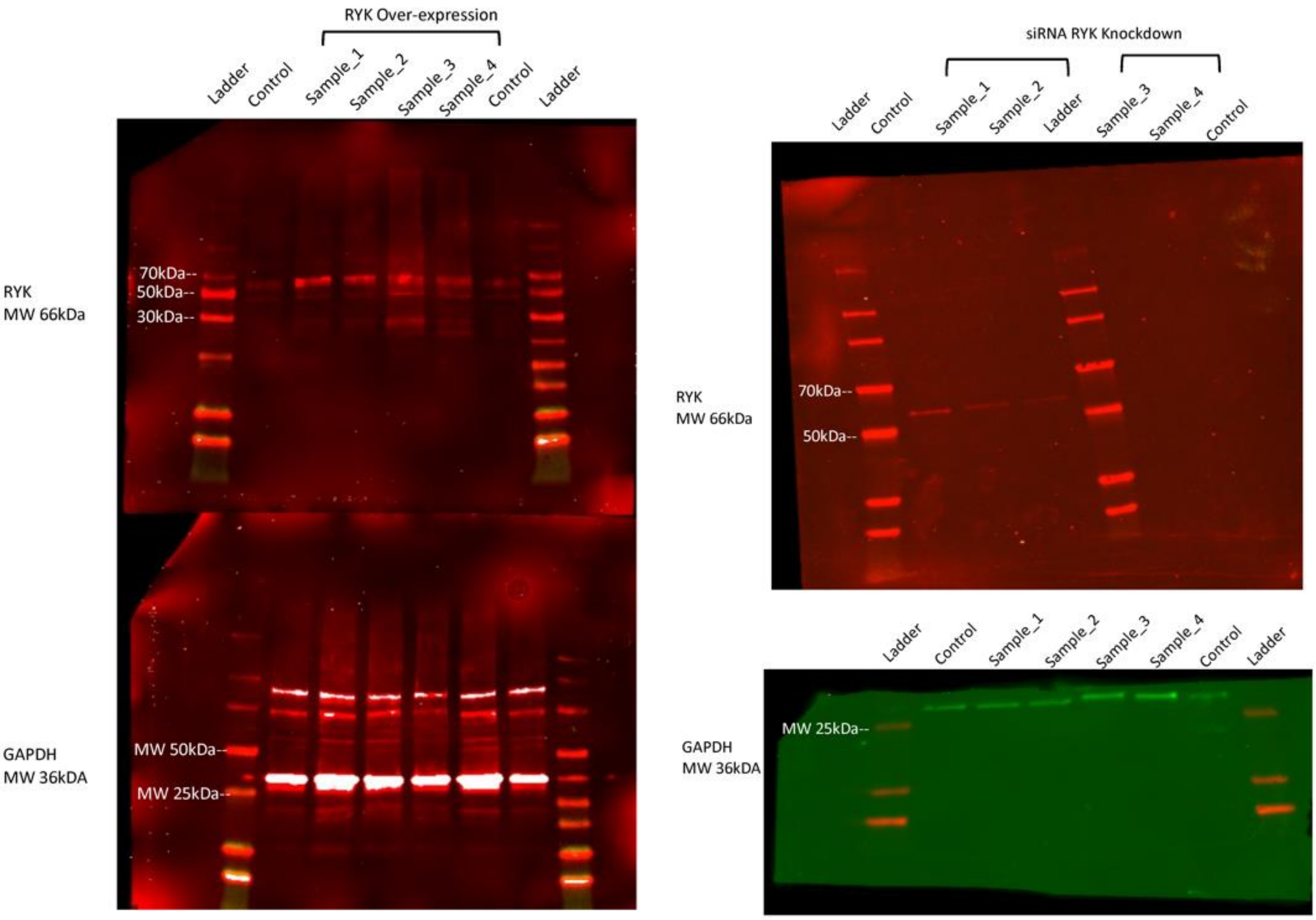

(**a**) (**b**)

Figure S5. Western blots. (a) HEK *RYK* overexpression and **(b)** knockdown.

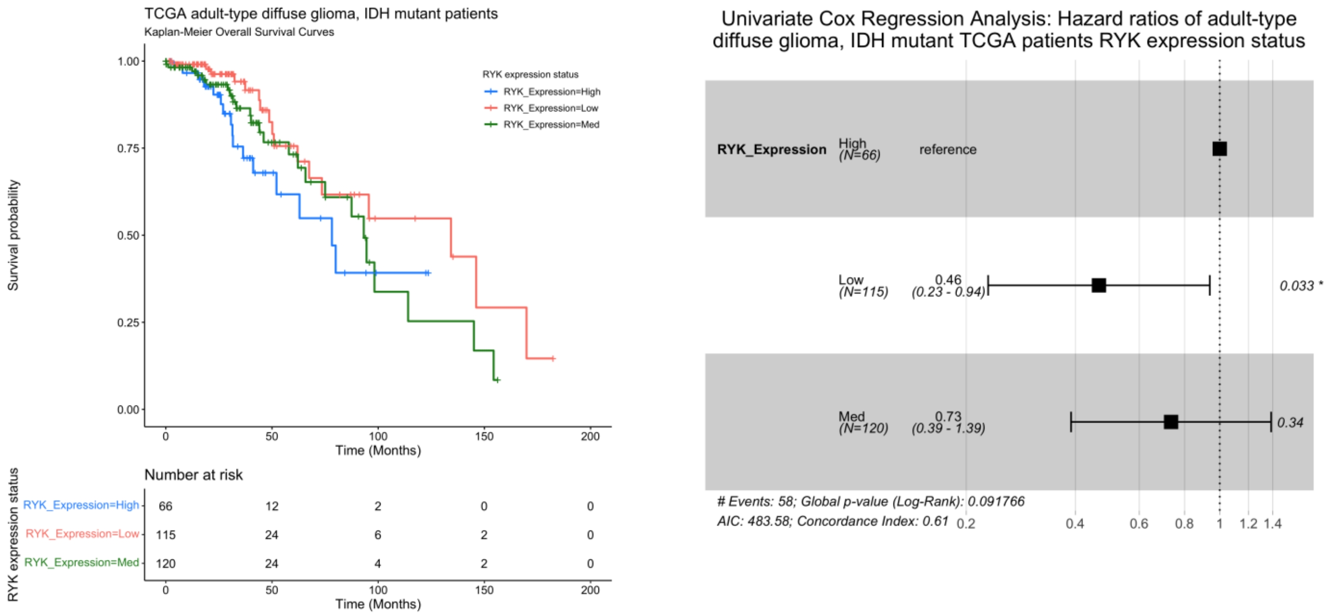

(a) (b)

Figure S6. Univariate Cox proportional hazards regression analysis of OS for the TCGA IDH mutant glioma patients' cohorts. (a) Kaplan-Meier curves illustrating the impact of *RYK* gene expression on OS.**(b)** Forest plot showing the HR and CI of *RYK* gene expression status.

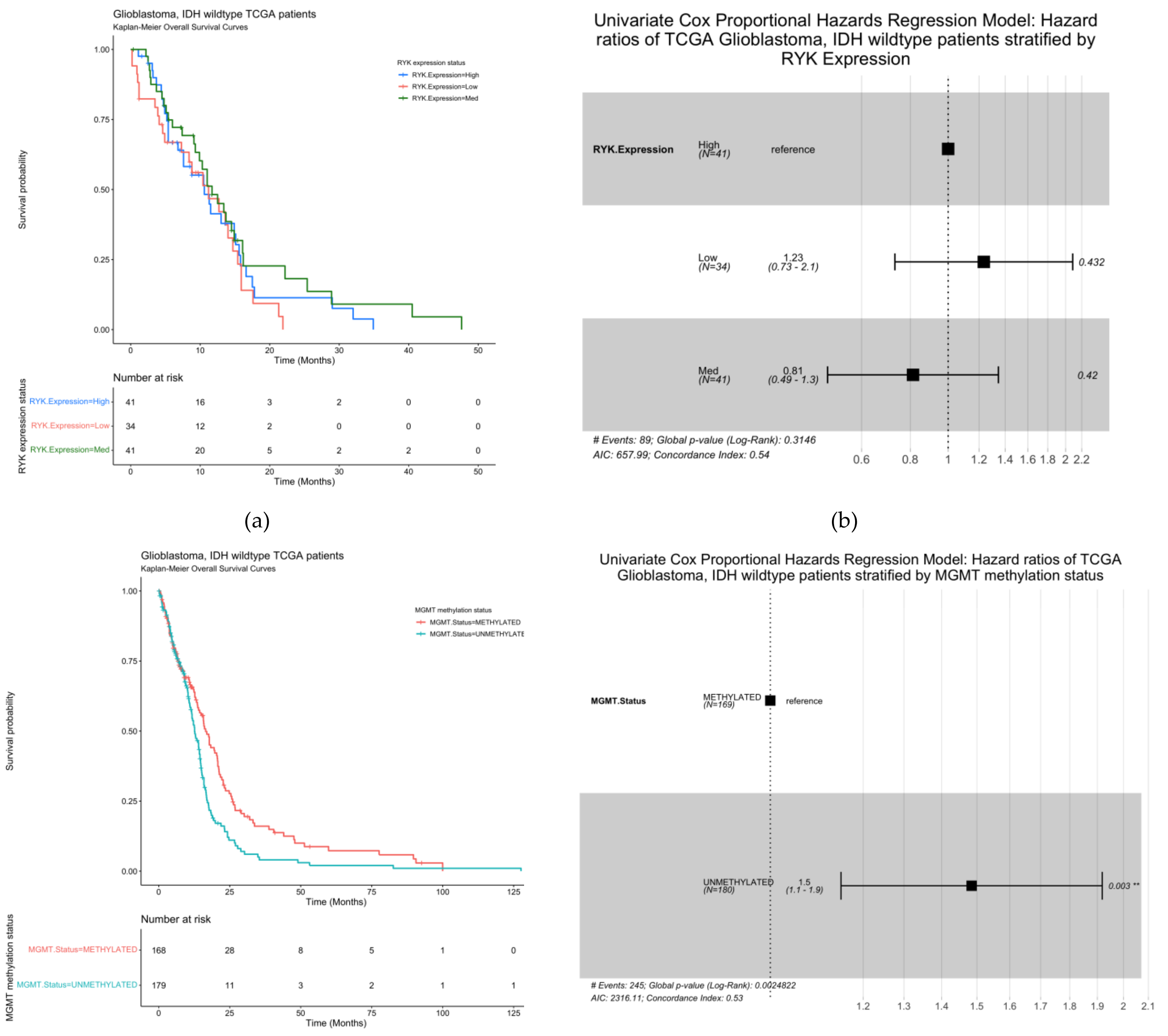

(c) (d)

Figure S7. Univariate Cox proportional hazards regression analysis of OS for the TCGA IDH wildtype GBM patients' cohorts.(a) Kaplan-Meier curves illustrating the impact of *RYK* gene expression on OS.**(b)** Forest plot showing the HR and CI of *RYK* gene expression status.**(c)** Kaplan-Meier curves illustrating the impact of *MGMT* methylation status on OS. **(d)** Forest plot showing the HR and CI of *MGMT* methylation status.

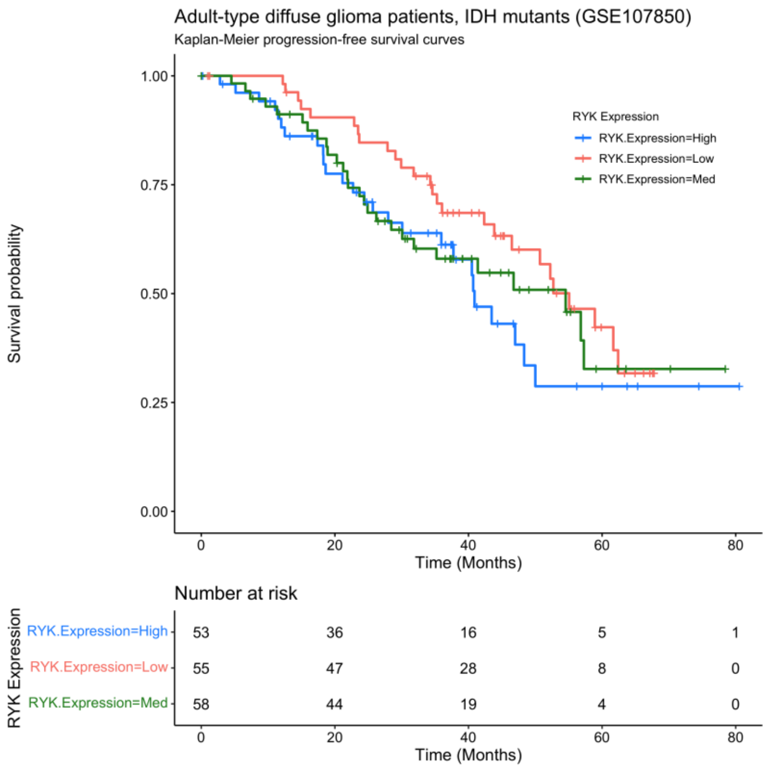

Figure S8. Univariate Cox proportional hazards regression analysis of OS for GSE1078 IDH mutant glioma with TMZ therapy patients' cohort.(a) Kaplan-Meier curves illustrating the impact of *RYK* gene expression on OS.

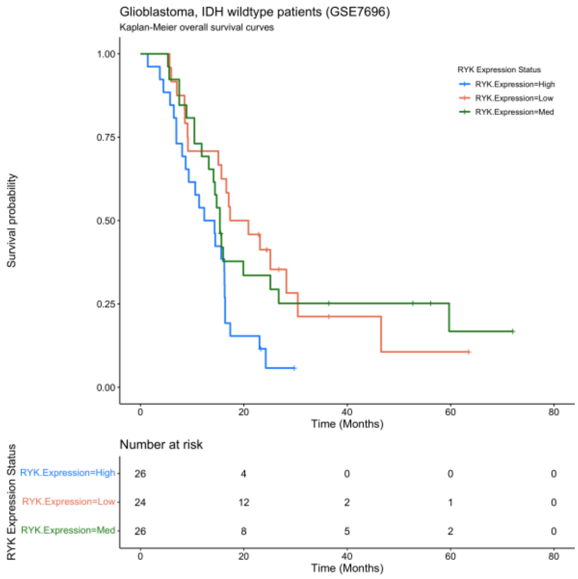
Glioblastoma, IDH wildtype patients (GSE7696)
Kaplan-Meier overall survival curves
Survival probability
RYK Expression Status
RYK.Expression=High
RYK.Expression=Low
RYK.Expression=Med
Time (Months)
Number at risk
RYK Expression Status
RYK.Expression=High 26 4 0 0 0
RYK.Expression=Low 24 12 2 1 0
RYK.Expression=Med 26 8 5 2 0
Time (Months)

(a)

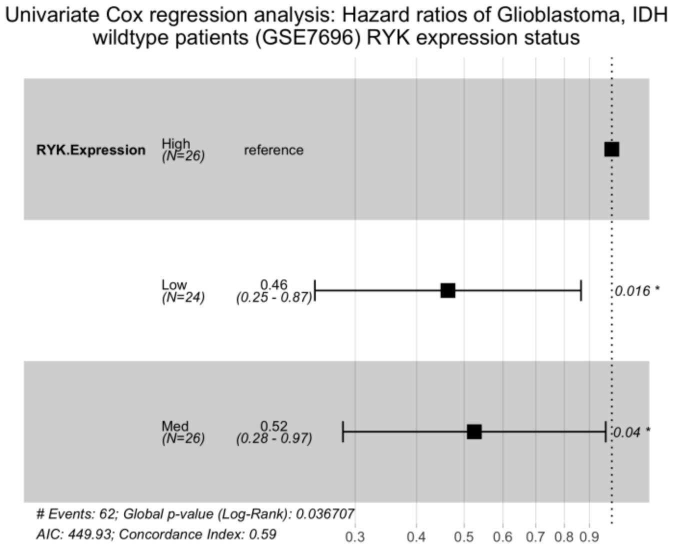
Univariate Cox regression analysis: Hazard ratios of Glioblastoma, IDH wildtype patients (GSE7696) RYK expression status
RYK.Expression High (N=26) reference
Low (N=24) 0.46 (0.25 - 0.87) 0.016 *
Med (N=26) 0.52 (0.28 - 0.97) 0.04 *
Events: 62; Global p-value (Log-Rank): 0.036707
AIC: 449.93; Concordance Index: 0.59
0.3 0.4 0.5 0.6 0.7 0.8 0.9

(b)

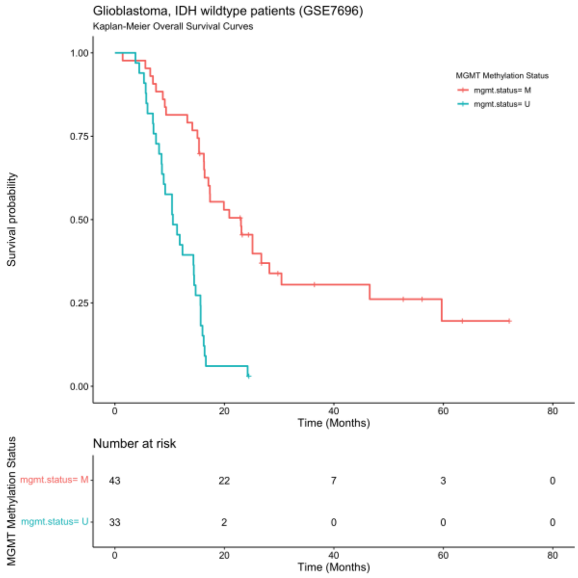
Glioblastoma, IDH wildtype patients (GSE7696)
Kaplan-Meier Overall Survival Curves
Survival probability
MGMT Methylation Status
mgmt.status= M
mgmt.status= U
Time (Months)
Number at risk
MGMT Methylation Status
mgmt.status= M 43 22 7 3 0
mgmt.status= U 33 2 0 0 0
Time (Months)

(c)

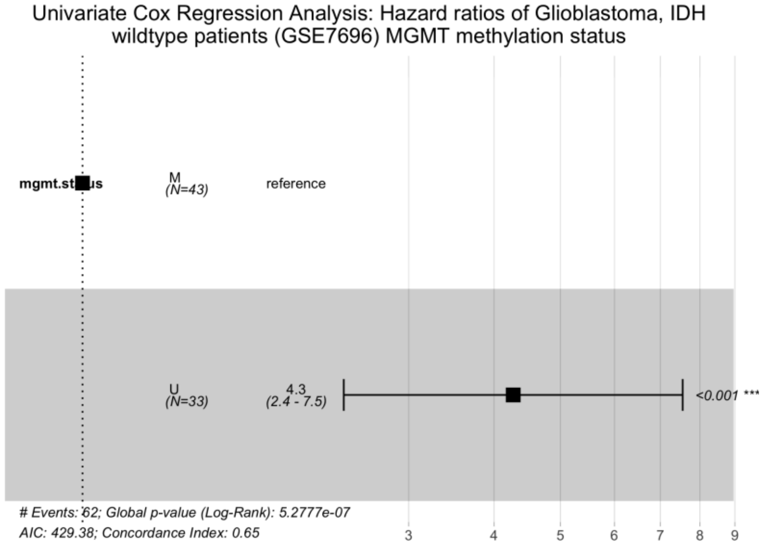
Univariate Cox Regression Analysis: Hazard ratios of Glioblastoma, IDH wildtype patients (GSE7696) MGMT methylation status
M (N=43) reference
U (N=33) 4.3 (2.4 - 7.5) <0.001 ***
Events: 62; Global p-value (Log-Rank): 5.2777e-07
AIC: 429.38; Concordance Index: 0.65
3 4 5 6 7 8 9

(d)

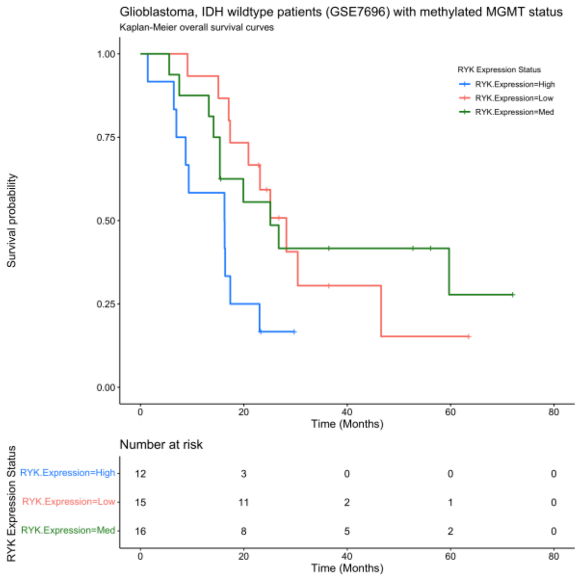

(e)

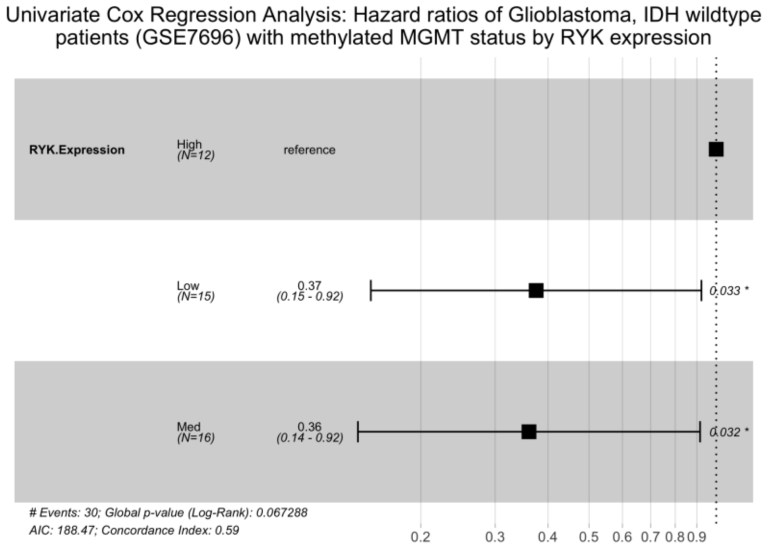

(f)

Figure S9. Univariate Cox proportional hazards regression analysis of OS GSE7696 IDH wildtype GBMs with TMZ therapy patients' cohort.(a) Kaplan-Meier curves illustrating the impact of *RYK* gene expression on OS.**(b)** Forest plot showing the HR and CI of *RYK* gene expression status.**(c)** Kaplan-Meier curves illustrating the impact of *MGMT* methylation status on OS. **(d)** Forest plot showing the HR and CI of *MGMT* methylation status.**(e)** Kaplan-Meier curves illustrating the impact of *RYK* expresion in *MGMT* methylated patients on OS.**(f)** Forest plot showing the HR and CI of of *RYK* expresion in *MGMT* methylated patients.
